# Testing the Feasibility of a Passive and Active Case Ascertainment System for Multiple Rare Conditions Simultaneously: The Experience in Three US States

**DOI:** 10.2196/publichealth.5516

**Published:** 2016-08-29

**Authors:** Amanda Reichard, Suzanne McDermott, Margaret Ruttenber, Joshua Mann, Michael G Smith, Julie Royer, Rodolfo Valdez

**Affiliations:** ^1^ Institute on Disability University of New Hampshire Durham, NH United States; ^2^ Department of Epidemiology and Biostatistics University of South Carolina Columbia, SC United States; ^3^ Special Health Care Needs Colorado Department of Public Health and Environment Denver, CO United States; ^4^ Department of Preventive Medicine University of Mississippi Medical Center Jackson, MS United States; ^5^ South Carolina Department of Health and Environmental Control Columbia, SC United States; ^6^ Revenue and Fiscal Affairs Office South Carolina Budget and Control Columbia, SC United States; ^7^ National Center for Birth Defects and Developmental Disabilities Centers for Disease Control and Prevention Atlanta, GA United States

**Keywords:** spina bifida, muscular dystrophy, fragile X syndrome, surveillance

## Abstract

**Background:**

Owing to their low prevalence, single rare conditions are difficult to monitor through current state passive and active case ascertainment systems. However, such monitoring is important because, as a group, rare conditions have great impact on the health of affected individuals and the well-being of their caregivers. A viable approach could be to conduct passive and active case ascertainment of several rare conditions simultaneously. This is a report about the feasibility of such an approach.

**Objective:**

To test the feasibility of a case ascertainment system with passive and active components aimed at monitoring 3 rare conditions simultaneously in 3 states of the United States (Colorado, Kansas, and South Carolina). The 3 conditions are spina bifida, muscular dystrophy, and fragile X syndrome.

**Methods:**

Teams from each state evaluated the possibility of using current or modified versions of their local passive and active case ascertainment systems and datasets to monitor the 3 conditions. Together, these teams established the case definitions and selected the variables and the abstraction tools for the active case ascertainment approach. After testing the ability of their local passive and active case ascertainment system to capture all 3 conditions, the next steps were to report the number of cases detected actively and passively for each condition, to list the local barriers against the combined passive and active case ascertainment system, and to describe the experiences in trying to overcome these barriers.

**Results:**

During the test period, the team from South Carolina was able to collect data on all 3 conditions simultaneously for all ages. The Colorado team was also able to collect data on all 3 conditions but, because of age restrictions in its passive and active case ascertainment system, it was able to report few cases of fragile X syndrome. The team from Kansas was able to collect data only on spina bifida. For all states, the implementation of an active component of the ascertainment system was problematic. The passive component appears viable with minor modifications.

**Conclusions:**

Despite evident barriers, the joint passive and active case ascertainment of rare disorders using modified existing surveillance systems and datasets seems feasible, especially for systems that rely on passive case ascertainment.

## Introduction

### Methods for Surveillance of Rare Conditions

Surveillance is used to gather data and knowledge that can be used to identify and control a health problem or to improve a public health program or service [1]. Birth defects monitoring programs, which focus primarily on identifying diagnosed cases, have widely used passive systems that gather data from administrative data such as hospital discharge records and administrative records from public insurers [2]. However, these systems can both over- and underestimate the actual prevalence of conditions, because coding for billing purposes is not always accurate or complete [3-5].

Active case ascertainment methods are considered the “gold standard” in public health surveillance [6]. These methods involve trained coders collecting data directly from medical providers, health service providers, and educational institutions to identify cases. The biggest challenge for using these methods is locating people with any of the conditions of interest within a designated geographic area [7]. Thus, population-based approaches must actively review records from diverse sources (eg, inpatient and outpatient settings, rehabilitation services, disability-specific programs, schools) [8].

Rare conditions such as spina bifida, fragile X syndrome, and muscular dystrophy can be especially difficult to monitor. Among these 3 conditions, only spina bifida is recognizable at birth and more easily included in state-based birth defects monitoring systems. The other 2 conditions—fragile X syndrome and muscular dystrophy—are not apparent in the early days of life and no laboratory test or biomarker is commonly used to screen newborns for these conditions. Instead, fragile X syndrome and muscular dystrophy are often identified during early childhood after parents and primary care providers note developmental or motor deficits. Both conditions require specific genetic tests to confirm their diagnosis; however, this usually occurs in an outpatient setting and does not require hospitalization. Thus, the data sources for passive surveillance of these conditions need to be extended not only to genetic laboratories but also to specialty care clinics where diagnosis is confirmed typically after multiple visits [9-11].

### Spina Bifida

Spina bifida occurs when the neural tube fails to close properly during fetal development [12-14]. The incidence of spina bifida detected at birth (namely, meningocele and myelomeningocele) decreased from approximately 2.5/10,000 (95% CI 2.3-2.7) in 1992 to 2.0/10,000 (95% CI 1.9-2.1) in 2001. The National Birth Defects Prevention Network estimated the *prevalence* of spina bifida (without hydrocephaly) was 3.5/10,000 live births (95% CI 3.31-3.68) [14].

### Muscular Dystrophy

Muscular dystrophies are a group of genetic diseases characterized by progressive skeletal muscle degeneration and weakness [15]. Although more than 30 forms of muscular dystrophy have been identified, there are 9 major forms [16]. The most common muscular dystrophies, Duchenne muscular dystrophy and Becker muscular dystrophy, together have an estimated prevalence of 1.38/10,000 males aged 5 to 24 years [16]. Other major forms (eg, distal, Emery-Dreifuss) each have a prevalence of 1 to 2 per 100,000 [17,18].

### Fragile X Syndrome

Fragile X syndrome results from a mutation in the fragile X mental retardation 1 gene, *FMR1*, on the X chromosome [19]. Impairment severity can range from relatively mild learning disabilities to moderate intellectual disability and autism or “autistic-like” behaviors. Approximately 1 in 3600 to 4000 males and 1 in 4000 to 6000 females is born with the full mutation for fragile X. The vast majority of males and about 50% of females with the full mutation will have fragile X syndrome [19-21].

### Importance of Surveillance of These 3 Rare Conditions

These conditions have low prevalence but a great impact on long-term disability, mortality, cost, and caregiver stress [12,22,23]. Obtaining a better estimate of state prevalence is a necessary starting point for assessing the impact. Such tracking requires the following: a flexible population scope (eg, specific to infants and young children); expansion of data sources (eg, health care specialists and tertiary medical centers); and labor-intensiveness (eg, data abstracted from a large number of health care practices).

Spina bifida, muscular dystrophy, and fragile X syndrome were chosen for this feasibility study because (1) they are all low incidence conditions with high health and economic impact; (2) they encompass a wide range of etiological, physical, and cognitive symptoms; (3) they represent various degrees of difficulty for passive and active case ascertainment; (4) public and private organizations have long-standing active research programs and data collections on these 3 conditions; and (5) the US Congress has provided special funding to monitor and study these 3 conditions [24-27].

The low prevalence of rare disorders makes impractical the development of a separate passive and active case ascertainment system for each condition but lends itself to a combined case ascertainment system that would monitor several conditions at once. Such an integrated passive and active case ascertainment system could serve as a model for other low prevalence and high-impact conditions. Simultaneous passive and active case ascertainment of rare conditions may lead to public health interventions that improve the health of a sizable segment of the population affected by these conditions. Thus, the purpose of this paper is to report on the feasibility of developing a rare conditions passive and active case ascertainment system that simultaneously monitors spina bifida, muscular dystrophy, and fragile X syndrome within a state. In each of the 3 states in which this work was undertaken, objectives were to (1) assess the ability of the local passive and active case ascertainment systems to capture the 3 rare disorders, (2) capture preliminary state prevalence estimates of the conditions, and (3) discover barriers and facilitators to such implementation. Findings can provide lessons for future rare conditions passive and active case ascertainment activities in states with similar systems to these.

## Methods

### Justification of States Included in the Study

The design of this feasibility study required that the states included were at varying levels of readiness toward implementing a rare conditions passive and active case ascertainment program. The state teams were selected by the study team, based on knowledge of states with disability epidemiologists and their understanding of data capability. States were also selected so that they roughly conformed to one each on the high, medium, and lower levels of passive and active case ascertainment sophistication. Colorado (high) and South Carolina (medium) were selected because of their established and state-supported birth defects passive and active case ascertainment system and, in the case of South Carolina, other integrated data system capacities. Kansas (low) was selected because it was home to a disability epidemiologist who had published articles using the Medicaid system to analyze disability and health issues. The approach in each state was to identify existing passive and active case ascertainment systems and data sources that could be expanded to implement a more comprehensive system within 2 years. The 2-year duration was chosen to allow up to 1 year for the assessment of current passive and active case ascertainment systems, standardization of case definitions, and selection of variables for the active case ascertainment component; and an additional year to test the feasibility of modestly enhancing the existing systems to assess what is feasible and to compare the three systems. Within each state, systems were compared by (1) using data from the active system to assess the accuracy of cases identified in the passive system and (2) capture-recapture methodology to get estimates of the prevalence of the 3 conditions.

The first step was to document how states varied in their different resources, approaches, and levels of experience in conducting passive and active case ascertainment. Table 1 compares passive and active case ascertainment programs in the 3 states [28,29]. The same sources and variables were searched and assessed for all 3 conditions.

Colorado has a mature, state-mandated, birth defects surveillance system in the health department, which requires reporting up to age 3 years. This system conducts active case finding through administrative data sources and it includes reports of spina bifida that mirror expected prevalence. Colorado also has a Centers for Disease Control and Prevention (CDC)–funded muscular dystrophy surveillance program (Muscular Dystrophy Surveillance, Tracking, and Research Network; MD STARnet) that uses active surveillance in neuromuscular clinics to identify childhood-onset cases. The MD STARnet system had been ongoing for 10 years at the beginning of our project. The challenge for Colorado was to identify cases of fragile X syndrome, because there had only been a few cases reported in the past decade. Fragile X syndrome is an inherited cause of intellectual disability, which is not apparent at birth. When intellectual disability is diagnosed in a child, a genetic diagnostic examination is needed to make the fragile x syndrome diagnosis; thus, in many cases this is not done before the child’s third birthday. The fact that Colorado only had a few cases of fragile X syndrome reported before the onset of the project suggests that even established birth defects systems need expanded authority through their legislature to collect information about people who are older than 3 years. The Colorado passive and active case ascertainment system needs to pursue sources of reporting, such as the fragile X clinic in their state, if they want to conduct passive and active case ascertainment of rare conditions. South Carolina has a birth defects case ascertainment system that relies on active hospital record abstraction for spina bifida and other early identifiable birth defects. This system had been collecting cases of spina bifida at the prevalence rate that was expected. South Carolina also has a well-established administrative data system that allows linkage among a large number of public insurance, vital records, and state service agency secondary data sources. This system was available for passive ascertainment. The South Carolina project developed an active surveillance system in 5 counties to compare the active and passive ascertainment of the 3 conditions. Kansas, likewise, has a birth defects surveillance system housed in the health department that primarily relied on birth certificate reporting of birth defects. This system has a passive case ascertainment component and no active case ascertainment component. Thus, the challenge for Kansas was to develop a small, active, case ascertainment component for this project.

During the first year of this 2-year project the teams from Colorado and South Carolina began with the identification of existing data sources. The Kansas team spent the first year cataloging features they could use from the other 2 states that had well-developed passive and active case ascertainment systems, investigating the capacity of local chapters of parent advocacy organizations for each condition, and conducting an extensive literature review. The Kansas team worked with the South Carolina and Colorado health departments to obtain information about policies and procedures related to existing passive and active case ascertainment systems, particularly on birth defects monitoring programs.

Together, the teams from the 3 states established uniform case definitions (including International Classification of Diseases, Ninth Revision, or ICD-9 codes and other required elements), variable definitions, and data abstraction tools for the active case ascertainment approach. The codes used in the 3 states are listed in Table 2. South Carolina and Kansas completed institutional review board (IRB) reviews that were needed because an active case ascertainment system that covered all ages was being used for this project, and this expanded an existing state statutory system.

**Table 1:** Major characteristics of the case ascertainment system in 3 US states.

Characteristics		Colorado	South Carolina	Kansas
**State characteristics**				
	Legislative authority	CRS^a^ 25-1.5-101 to 25-1.5-105; enacted in 1985	A281,R308,H4115; enacted in 2004	KSA^b^ 65-1241 to 65-1246; enacted in 2004
	Location	Department of Health: Epidemiology and Environment	Department of Health: Maternal and Child Health	Department of Health: Vital Statistics, Maternal and Child Health
	Characterization of the passive and active case ascertainment system	Mostly passive	Passive and active	Passive only
**Data characteristics**				
	Data sources	Case ascertainment (active, passive); vital records (birth, death, and fetal death certificates); state-based registries; delivery hospitals; pediatric and tertiary care hospitals; genetic laboratories; genetic counseling services; genetic clinics; physician reports.	Case ascertainment (active); vital records (birth, death, fetal death, and elective termination certificates); state-based registries; delivery hospitals; pediatric and tertiary hospitals; prenatal diagnosis facilities; genetic laboratories; genetic counseling services; genetic clinics; physician reports; passive sources; Medicaid; hospital discharges; state health plan claims; Department of Disabilities and Special Needs.	Case ascertainment (passive); vital records (birth and fetal death certificates); state-based registries; physician reports.
	Time frame—years covered for passive case ascertainment	SB^c^: 2010-12 MD^d^: 1992-2011 FXS^e^: 2007-12	SB: 1996-2012 MD: 1996-2012 FXS: 1996-2012 (secondary data with code)	SB: 1979-2013
	Time frame—years covered for active case ascertainment	SB: 2010-12 MD: 1992-2011 FXS: 2007-12	SB: 2013-2014 MD: 2013-2014 FXS: 2013-2014 (for prevalent cases)	SB: 1971-2013
	Age range covered, years	0-3 (SB, FXS); 0-28 (MD)	No limit	No limit
	Data sources for the active case ascertainment component in this project	Reascertainment and medical records.	Medical records (5 counties).	Medical records (1 county).
	Clinical review for the passive component of this project	Yes	No	No
	Clinical review for the active component of this project	Yes	Yes	Yes
	Barriers to implementation	Not enough time to change the age of reporting FXS from 3 to 10 years.	The active component could be labor intensive.	Limited availability and interconnection of data sources.

^a^CRS: Colorado Revised Statutes.

^b^KSA: Kansas Statutes Annotated.

^c^SB: spina bifida.

^d^MD: muscular dystrophy.

^e^FXS: fragile X syndrome.

**Table 2:** Diagnosis codes and variables used to passively identify cases in a pilot project for a 3-state (Colorado, Kansas, South Carolina) public health passive and active case ascertainment system for 3 rare conditions (spina bifida, muscular dystrophy, and fragile X syndrome).

Condition	Hospital discharge and insurance	Birth certificates	Death certificates
	ICD-9-CM^a^ all diagnosis fields	Source-specific indicator	ICD-10^b^ all cause fields
Fragile X syndrome	759.83	N/A^c^	Q99.2
Muscular dystrophy	359.0, 359.1, and 359.21	N/A	G71.0 and G71.1
Spina bifida (without anencephalies)	741.0 through 741.9	Myelomeningocele or meningocele variable	Q05 including Q05.0 to Q05.9

^a^ICD-9-CM: International Classification of Diseases, Ninth Revision, Clinical Modification.

^b^ICD-10: International Classification of Diseases, Tenth Revision.

^c^N/A: not applicable.

The teams assessed the status of statewide data sources for rare conditions passive and active case ascertainment in their respective states. All 3 states have birth defects registries that obtain data from birth certificates. Colorado and South Carolina queried electronic administrative data sources, including hospital and emergency department encounters, physician office visits, all of which include codes for conditions, services, and charges, as well as death records.

During the second year the states carried out both active and passive case ascertainment, documented barriers and challenges as they arose, and made tentative estimations of the prevalence of spina bifida, muscular dystrophy, and fragile X syndrome in each state.

### Methods by State

Each state’s methodology was based on its readiness for combined passive and active case ascertainment, the organization that led the project, the type of available data, and the answers to unanticipated barriers to the implementation of the passive and active case ascertainment system. The Guidelines for Conducting Birth Defects Surveillance [24] was the document that set the standards for operations in both Colorado and South Carolina and it was used to establish activities during this feasibility project.

#### Colorado

The Colorado Department of Public Health and Environment has a sophisticated, mainly passive, birth defects case ascertainment system that interfaces with some administrative data sources. The state has more than 20 years of surveillance data collection, under statutory authority, on a large array of childhood conditions, which include active case ascertainment data for muscular dystrophy and spina bifida.

##### Colorado’s Approach to Passive Case Ascertainment for Persons With Fragile X Syndrome, Spina Bifida, and Muscular Dystrophy

Colorado Responds to Children with Special Needs (CRCSN) is the program in charge of birth defects monitoring and prevention in the state. This program used case reports from multiple external sources to ascertain cases of each of the 3 conditions of interest among children from birth to age 3 years, for all cases meeting criteria. Case reports were entered into a transitional computer program that prepared the case for further processing before being posted to a core database. All case reports went through an extensive search and/or match process that linked cases to a unique identifier.

Colorado has participated in a number of CDC-funded surveillance efforts that have enhanced its data collection processes. For example, CRCSN collected data on children with spina bifida up to age 3 years through the CDC-funded Rapid Ascertainment project that uses a passive registry followed by a reascertainment and medical record review as confirmation for all live births [30]. Colorado has also participated in the CDC-funded MD STARnet program since it began surveillance for Duchenne and Becker muscular dystrophies in 2002 [31]. Recently, MD STARnet was expanded to include 7 additional types of muscular dystrophy. CRCSN has collected information on fragile X syndrome since 1994, through its passive Birth Defects Monitoring system, but only a few cases were reported in recent years. As part of this feasibility passive and active case ascertainment effort the Colorado project staff met with the director of the fragile X clinic at Children’s Hospital Colorado in an effort to expand the passive ascertainment and explore the possibility of conducting a medical record review to identify persons for whom a genetics laboratory confirmation was available. This contact was necessary to inform the director of the clinic’s responsibility to report children younger than 3 years with fragile X syndrome to the CRCSN system.

##### Colorado’s Approach to Active Case Ascertainment of Persons With Fragile X Syndrome, Spina Bifida, and Muscular Dystrophy

The CRCSN staff established specific guidelines for each case definition, which required the number of times a diagnosis is reported, the number of reporting sources, and the use of International Classification of Diseases, Ninth Revision, Clinical Modification (ICD-9-CM) diagnosis and procedure codes. The CRCSN did not implement an active case ascertainment system per se; instead, it supplemented the passive system with rigorous reascertainments and medical record reviews by physicians of all spina bifida cases and some muscular dystrophy cases. Before this feasibility project, this approach was not used for cases of fragile X syndrome.

CRCSN has developed a multistep approach to monitor data sources, specific diagnoses, over- and under-ascertainment, and problematic code assignment issues. Its data quality validation procedure includes computerized and manual approaches (computer screen review of case records, medical records review of selected cases, and clinical consultation when difficulties were identified). Additionally, two levels of clinical reviews are used for diagnosis: (1) staff level (to identify problems in report date, source of diagnosis, site of the encounter, and ICD-9-CM codes); (2) medical specialist level (to review the medical records and other relevant information to verify the diagnosis).

#### South Carolina

In this state, faculty from the University of South Carolina School of Public Health and the Medical University of South Carolina partnered with the South Carolina Department of Health and Environmental Control (DHEC) to complete this project. The active case ascertainment was conducted by the DHEC and the data utilized for the passive case ascertainment system was housed within the Revenue and Fiscal Affairs (RFA) agency. The RFA has agreements with state agencies and organizations to store data, although each data source retains control of the data at all times. Thus, RFA facilitated the development of written agreements with each agency and organization that potentially had data elements that could be used for this project. For deidentified data projects, including this rare conditions passive and active case ascertainment effort, the RFA uses an algorithm that relies on source-specific personal identifiers to create a global unique identifier. The data are edited and standardized before being run through the search algorithm. The global unique identifier is not associated with any personal identifier and is used on all subsequent episodes of services, regardless of data source or service provider. Using the unique global identifier enables staff to use data across multiple providers while protecting confidentiality. The investigators of the rare conditions passive and active case ascertainment system included one RFA staff member, a health department investigator, and university investigators.

##### South Carolina’s Approach to Passive Case Ascertainment of Persons With Fragile X Syndrome, Spina Bifida, and Muscular Dystrophy

All persons with an ICD-9-CM primary or secondary diagnosis code for fragile X syndrome, spina bifida, or muscular dystrophy were identified from Medicaid, the Hospital Discharge Dataset, and the claims from the State Health Plan that insures all government workers and their families (Table 2). The South Carolina DHEC Birth Defects Monitoring Program was used to identify cases of spina bifida. For all 3 conditions, death certificate records were used to identify unique cases from previous periods. These cases occur if people received care out of state or if they did not have their condition diagnosis included when they were seen in the medical care system. These cases were identified by International Classification of Diseases, Tenth Revision, (ICD-10) cause-of-death codes for fragile X syndrome, spina bifida, and muscular dystrophy from the South Carolina DHEC. For fragile X syndrome and muscular dystrophy, South Carolina also identified cases through the Department of Disabilities and Special Needs, the agency that provides support and services to people with disability.

##### South Carolina’s Approach to Active Case Ascertainment of Persons With Fragile X Syndrome, Spina Bifida, and Muscular Dystrophy

South Carolina DHEC established an active case ascertainment component for citizens of all ages with rare conditions in 5 South Carolina counties for this project, after IRB approval. After cases were reported to the South Carolina DHEC Birth Defects Monitoring Program by the physician practices, a registered nurse abstractor traveled to each practice to abstract relevant data (ie, basic diagnosis and demographic information; sufficient but less rigorous than the Colorado MD STARnet protocol for muscular dystrophy) from medical records of those who had received treatment in a clinic, hospital, or practice located in those 5 counties. The data were then entered into an Epi Info [32] data system for compilation, output into an SAS [33] file for editing, linking with passive data, and analysis.

##### South Carolina’s Approach to Merging the Passive and Active Systems

At the conclusion of active data collection for the 5 designated counties, the data from the passive case ascertainment system and the active case ascertainment system were linked and analyzed at RFA. The data collected through the passive system were compared with the data from the active case ascertainment system to test the accuracy and completeness of the passive system. Non-RFA investigators were provided with aggregate reports.

#### Kansas

Congenital anomalies have been recorded in Kansas birth certificates since 1979 and since 1982, Kansas has had, under administrative regulations, a limited set of passive case ascertainment activities for these anomalies [28]. In 2004, the Kansas legislature issued the statutes for the creation of the Birth Defects Information System (BDIS) with the aim of collecting information on congenital anomalies and other birth abnormalities among children younger than 5 years [28]. Currently, the BDIS includes an interface between a birth defects database and a Web-based application from the program Children and Youth with Special Health Care Needs, along with relevant variables from the Vital Statistics Integrated Information System. Thirteen anomalies are currently listed in the birth certificates and reported to the BDIS [28].

##### Kansas’ Approach to Passive Case Ascertainment for Persons With Fragile X Syndrome, Spina Bifida, and Muscular Dystrophy

Given the strong reliance of the BDIS on birth certificates and because spina bifida is the only one of the 3 conditions included in this report that is diagnosed at birth, the Kansas team developed a passive case ascertainment plan just for spina bifida. First, the team identified cases of spina bifida using ICD-9-CM codes (see Table 2) in a Medicaid claims database. Then, relevant individual information from these cases was merged with individual information from the BDIS. Finally, the individual data were deduplicated and aggregated to obtain the count of spina bifida cases in the state.

##### Kansas’ Approach to Active Case Ascertainment for Persons With Fragile X Syndrome, Spina Bifida, and Muscular Dystrophy

For the active case ascertainment component, the Kansas team mailed a letter to pediatricians, neurologists, and spina bifida clinics in one county (Sedgwick) in Kansas asking them to report all of their cases of spina bifida, under authority of the Kansas Birth Defects Act (KSA 65-1241 through 65-1246). Sedgwick County, including the city of Wichita, is a large county, including 17.5% of the state’s total population [34]. For professional medical record abstraction, eligible medical records were sent to the Kansas Foundation for Medical Care (KFMC), a state not-for-profit organization that has served as the state’s External Quality Review Organization and information technology resource in the state. Additionally, KFMC provided advice on data aggregation.

##### Kansas’ Approach to Merging the Passive and the Active Case Ascertainment Systems

When the active component of the project was completed, KFMC shared the active case ascertainment dataset with the Kansas team. These data were merged with the passive dataset to identify the cases that were common between both datasets.

## Results

### Findings by State

Within the 2-year period allocated to this project, the South Carolina team was able to use the passive and active case ascertainment capabilities of the state to assemble a passive and active case ascertainment system, with both passive and active components, to simultaneously detect spina bifida, muscular dystrophy, and fragile X syndrome. The active component of the South Carolina team, however, was limited to 5 counties. The Colorado team supplemented their extensive passive system with reascertainment and clinical review of the cases; however, because of age restrictions in reporting, this team was able to detect only a handful of cases of fragile X syndrome. The Kansas team was able to assemble a case ascertainment system with passive and active components, but only for 1 condition, spina bifida, and the active component of this system was limited to 1 county.

On the basis of cases found, the teams calculated number and prevalence for their respective states. These estimates are tentative, not comparable in any way from one state to the other, and certainly not comparable to published estimates calculated with more rigorous methods. Table 3 presents the data collected in the 3 states, collected for spina bifida, muscular dystrophy, and fragile X syndrome. The racial composition of cases in the 3 states differed substantially, but this composition follows the underlying population of each state. In 2014, a total of 66% of the South Carolina population was white, 27% black, and 5% Hispanic, and the cases of spina bifida and muscular dystrophy reflect this pattern. The cases of fragile X syndrome are 47% white for active and 54% for passive. In Colorado and Kansas, there were high proportions of missing data or “other” racial groups noted. The male to female ratio for spina bifida was not consistent across the 3 areas. More females than males were identified in South Carolina and more males than females were identified in both Colorado and Kansas. The higher proportion of male cases identified with muscular dystrophy and fragile X syndrome in Colorado and South Carolina (muscular dystrophy only) reflects the fact that these 2 conditions have genetic X-linked inheritance.

Table 4 presents the rates of spina bifida, muscular dystrophy, and fragile X syndrome identified in this pilot project. In Colorado, the rate for spina bifida was 3.35/10,000 for those younger than 3 years, for muscular dystrophy the rate was 1.29/10,000, and with only 6 identified cases of fragile X syndrome the rate was close to zero. The passive and active case ascertainment efforts in South Carolina were for all ages, so the denominator for South Carolina was the state population for passive case ascertainment and the all age population of 5 counties around the metropolitan areas of Columbia and Charleston for active case ascertainment. The South Carolina passive rates were substantially higher than active rates for spina bifida (2.16/10,000 for active and 12.15/10,000 for passive) and muscular dystrophy (3.28/10,000 for active and 6.84/10,000 for passive). For fragile X syndrome these rates were more similar, 1.20/10,000 for active and 1.65/10,000 for passive case ascertainment. In Kansas, for the one county used for active case ascertainment of spina bifida the rate was 0.86/10,000 and the passive rate was 3.04/10,000.

**Table 3:** Summary of data collected in a pilot project for a 3-state (Colorado, Kansas, South Carolina) public health passive and active case ascertainment system for 3 rare conditions (spina bifida, muscular dystrophy, and fragile X syndrome).

Condition		Colorado^a^ (64 counties) n (%)	South Carolina active (5 counties) n (%)	South Carolina passive (46 counties) n (%)	Kansas active (1 county) n (%)	Kansas passive (statewide) n (%)
Spina bifida, N		...^b^	253	5872	...	882
	Non-Hispanic black	...	73 (28.8)	1590 (27.1)	...	25 (2.8)
	Non-Hispanic white	30 (...)	138 (54.5)	3628 (61.8)	21 (...)	296 (33.6)
	Hispanic and others	28 (...)	42 (16.6)	654 (11.1)	19 (...)	561 (63.6)
	Male	36 (61.0)	112 (44.3)	2266 (38.6)	26 (59.1)	392 (55.6)
	Female	10 (16.9)	141 (55.7)	3606 (61.4)	18 (40.9)	490 (44.4)
	Missing	13 (22.0)				
	Birth year, range and 95% CI	Range 2010-2012	95% CI 1982-2014	95% CI 1958-1998	Range 1971-2013	Range 2004-2013
Muscular dystrophy^c^, N		689	384	3305		
	Non-Hispanic black	16 (2.3)	75 (19.5)	805 (24.4)		
	Non-Hispanic white	379 (55.0)	262 (68.2)	1998 (60.5)		
	Hispanic and others	294 (42.7)	47 (12.2)	502 (15.2)		
	Male	444 (64.4)	258 (67.2)	1707 (51.6)		
	Female	245 (35.6)	126 (32.8)	1598 (48.4)		
	Birth year, range and mean (SD)	1992-2011	1973 (23)	1969 (24)		
Fragile X syndrome, N		6	141	795		
	Non-Hispanic black		57 (40.4)	286 (36.0)		
	Non-Hispanic white		66 (46.8)	433 (54.5)		
	Hispanic and others		18 (12.7)	76 (9.6)		
	Male		88 (62.4)	516 (64.9)		
	Female		53 (37.6)	279 (35.1)		
	Birth year, range and mean (SD)	2007-2012	1983 (19)	1981 (19)		

^a^Colorado did not implement an active case ascertainment system per se; instead, it supplemented the passive system with reascertainments and medical record reviews of all spina bifida cases and some muscular dystrophy cases.

^b^Ellipses indicate that the cells contain less than 5 individuals; owing to confidentiality concerns, the exact number has been suppressed.

^c^South Carolina and Colorado differed in the rigor of the active case ascertainment. Colorado, as part of Centers for Disease Control and Prevention’s Muscular Dystrophy Surveillance, Tracking, and Research Network (MD STARnet), used a very thorough protocol for active case ascertainment. South Carolina was less intensive, recording fewer key variables. Both recorded counts for the 9 major forms of muscular dystrophy. Kansas did not collect data on Muscular Dystrophy or Fragile X.

**Table 4:** Prevalence rates of spina bifida, muscular dystrophy, and fragile X syndrome in the 3 states, based on reference population.

Measure	South Carolina	Colorado	Kansas
State and subarea population used for rate calculation (2014)	4,832,482 state population for passive 1,170,249 for 5 counties^a^ for active case ascertainment	176,169 state for younger than 3 years	2,904,021 state population for passive 508,803 for 1 county for active case ascertainment
Rate of spina bifida	2.16/10,000 active 12.15/10,000 passive	3.35/10,000 younger than 3 years	0.86/10,000 active^b^ 3.04/10,000 passive
Rate of muscular dystrophy	3.28/10,000 active 6.84/10,000 passive	1.29/10,000	N/A^c^
Rate of fragile X syndrome	1.20/10,000 active 1.65/10,000 passive	0.01/10,000	N/A

^a^ The 5 South Carolina counties for active case ascertainment are as follows: Berkeley, Dorchester, Charleston, Lexington, and Richland.

^b^ Only 1 Kansas county was included in active case ascertainment.

^c^ N/A: not applicable.

### Barriers to Implementation

The Colorado team reported no major problems implementing this project as they already actively ascertained or reascertained from passive reports the cases of muscular dystrophy and spina bifida. The limited time for this project (2 years) did not allow Colorado to officially request approval from the state legislature to change the age of reporting for fragile X syndrome from 3 to 10 years, as has been done with autism and fetal alcohol syndrome. Therefore, Colorado did not have any prevalent cases of fragile X syndrome to report and only 6 incident cases.

The major barrier to implementing a combined passive and active case ascertainment system for rare disorders in South Carolina is cost. Passive data sources are readily available and the active case ascertainment system is a natural extension of the ongoing DHEC Birth Defects Monitoring Program; however, the incremental cost of expanding active case ascertainment can be substantial. For this project, a full-time registered nurse with previous active case ascertainment experience was hired. The costs for the project included her salary and fringe benefits, training and travel, the customary clinical review of cases found, and the storage and protection of data. Finally, the utility of identifying and monitoring new cases needs to be justified with clear benefits for the patients and their caregivers.

In Kansas, the BDIS relies heavily on data from birth certificates with the age of 5 years being the limit for reporting genetic or congenital conditions [28,29]. The monitoring of conditions that are detectable long after birth under such constraints, such as fragile X syndrome and muscular dystrophy, could be challenging. After limiting the test to spina bifida, a major policy barrier surfaced. The Kansas law that created BDIS also made the records contained in this system strictly confidential and disclosable only by court order. Public disclosure is only allowed in summary or aggregated formats (law identified in Table 1); therefore, merging, matching, and analyzing the data had to be performed in situ by team members affiliated with the Kansas Department of Health and Environment.

To facilitate data sharing regarding medical records, the Kansas team contacted KFMC to perform medical record abstraction for this project. The KFMC has an established and credible relationship with health care providers in Kansas and recently partnered with the Kansas Department of Health and Environment to complete a record review project on their behalf. However, even with this established relationship and the state law, there was a limited response from providers to participate in the active component.

## Discussion

This passive and active case ascertainment project was carried out to test the feasibility of establishing rare conditions passive and active case ascertainment systems for more than one condition at a time in states with varying levels of existing infrastructure. Having states at various stages of readiness for passive and active case ascertainment in this project allows for the identification of factors that may facilitate or impede the development of such systems.

The approach was to first assess the existing birth defects monitoring system in all 3 states, and then to identify additional processes that could be used to implement an ongoing rare conditions passive and active case ascertainment system. This project was not designed to compare active with passive case ascertainment; rather, it was designed to assess the feasibility of combining both types of case ascertainment systems to increase the yield of cases. The early steps for this project were successful: separately, all teams from the 3 states identified local data sources and determined the data elements to be collected. Together, the teams worked on the standardization of variable definitions to make the results comparable across systems and states. The definitions of the variables and their connections to the data sources were precisely documented to assure accurate replication.

This feasibility exercise helped us better understand the ways that states approach passive and active case ascertainment. Some states, like South Carolina and Colorado, have legal authority and ample experience monitoring birth defects. These 2 states use a variety of data sources for their passive and active case ascertainment systems, but active case ascertainment of rare diseases relies on obtaining funding through national programs, such as MD STARnet. In fact, after completion of this project, South Carolina was able to apply and get funding to join the national MD STARnet network. On the other hand, for other states, such as Kansas, the birth defects monitoring may have limited data sources and the implementation of a passive and active case ascertainment system to monitor more than one rare condition at once with both passive and active components would be a major challenge. Thus, as has been seen with other passive and active case ascertainment systems, funding through national networks or advocacy foundations appears to be the most viable approach to support the establishment of passive and active case ascertainment of rare conditions [35].

Because the purpose of the project was to assess the feasibility of establishing active and passive case ascertainment for rare conditions, we cannot guarantee the accuracy of the prevalence estimates; we have presented them here to provide reference for future work. Although the rates are not directly comparable because of state-to-state variations in data sources, prevalence of spina bifida was 2.16/10,000 people in South Carolina (active case ascertainment component), 3.35/10,000 in Colorado (passive case ascertainment component supplemented with case confirmations and clinical reviews), and 3.04/10,000 in Kansas (passive case ascertainment component; active case ascertainment resulted in limited follow-up). The prevalence of muscular dystrophy differed between South Carolina (3.28/10,000) and Colorado (1.29/10,000), although South Carolina used less rigorous criteria than the MD STARnet criteria and process [32] used by Colorado staff to define 9 forms of the condition. South Carolina was the only state able to test a combined passive and active case ascertainment approach for fragile X syndrome; the prevalence was 1.20/10,000 for active case ascertainment and 1.65/10,000 for passive case ascertainment.

The lessons learned have been valuable for the 3 states participating in this pilot project. Team participation and problem-solving approaches were excellent; however, state health departments will face obstacles if they seek to implement a combined passive and active case ascertainment system to simultaneously track rare disorders statewide. Although joint passive and active case ascertainment of rare disorders is feasible, only the passive component of case ascertainment seems to be readily available for use, even with limitations regarding data collection. The incorporation of the active component appears to be more of a long-term goal.

## Abbreviations

BDIS: birth defects information system
CDC: Centers for Disease Control and Prevention
CRCSN: Colorado Responds to Children with Special Needs
DHEC: Department of Health and Environmental Control
ICD-9: International Classification of Diseases, Ninth Revision,
ICD-9-CM: International Classification of Diseases, Ninth Revision, Clinical Modification
ICD-10: International Classification of Diseases, Tenth Revision,
IRB: institutional review board
KFMC: Kansas Foundation for Medical Care
MD STARnet: Muscular Dystrophy Surveillance, Tracking, and Research Network
RFA: Revenue and Fiscal Affairs
